# Whole-Genome Sequence of Multidrug-Resistant Methicillin-Resistant *Staphylococcus* *epidermidis* Carrying Biofilm-Associated Genes and a Unique Composite of SCCmec

**DOI:** 10.3390/antibiotics11070861

**Published:** 2022-06-24

**Authors:** Hisham N. Altayb, Hana S. Elbadawi, Othman Baothman, Imran Kazmi, Faisal A. Alzahrani, Muhammad Shahid Nadeem, Salman Hosawi, Kamel Chaieb

**Affiliations:** 1 Department of Biochemistry, Faculty of Science, King Abdulaziz University, Jeddah 21589, Saudi Arabia; oabaothman@kau.edu.sa (O.B.); ikazmi@kau.edu.sa (I.K.); faahalzahrani@kau.edu.sa (F.A.A.); mhalim@kau.edu.sa (M.S.N.); shosawi@kau.edu.sa (S.H.); kalshaib@kau.edu.sa (K.C.); 2 Centre for Artificial Intelligence in Precision Medicine, King Abdulaziz University, Jeddah 21589, Saudi Arabia; 3 Microbiology and Parasitology Department, Soba University Hospital, University of Khartoum, Khartoum 11115, Sudan; hanasalah200@gmail.com; 4 Laboratory of Analysis, Treatment and Valorization of Pollutants of the Environmental and Products, Faculty of Pharmacy, University of Monastir, Monastir 5000, Tunisia

**Keywords:** coagulase-negative *S. epidermidis*, MDR, SCCmec elements, *mecA*, CoNS

## Abstract

*Staphylococcus epidermidis* is part of the normal human flora that has recently become an important opportunistic pathogen causing nosocomial infections and tends to be multidrug-resistant. In this investigation, we aimed to study the genomic characteristics of methicillin-resistant *S. epidermidis* isolated from clinical specimens. Three isolates were identified using biochemical tests and evaluated for drug susceptibility. Genomic DNA sequences were obtained using Illumina, and were processed for analysis using various bioinformatics tools. The isolates showed multidrug resistance to most of the antibiotics tested in this study, and were identified with three types (III(3A), IV(2B&5), and VI(4B)) of the mobile genetic element SCCmec that carries the methicillin resistance gene (*mecA*) and its regulators (*mecI* and *mecR1*). A total of 11 antimicrobial resistance genes (ARGs) was identified as chromosomally mediated or in plasmids; these genes encode for proteins causing decreased susceptibility to methicillin (*mecA*), penicillin (*blaZ*), fusidic acid (*fusB*), fosfomycin (*fosB*), tetracycline (*tet*(*K*)), aminoglycosides (*aadD*, *aac*(*6′*)*-aph*(*2′’*)), fluoroquinolone (MFS antibiotic efflux pump), trimethoprim (*dfrG*), macrolide (*msr*(*A*)), and chlorhexidine (*qacA*)). Additionally, the 9SE strain belongs to the globally disseminated ST2, and harbors biofilm-formation genes (*icaA*, *icaB*, *icaC*, *icaD,* and IS256) with phenotypic biofilm production capability. It also harbors the fusidic acid resistance gene (*fusB*), which could increase the risk of device-associated healthcare infections, and 9SE has been identified as having a unique extra SCC gene (*ccrB4*); this new composite element of the *ccr* type needs more focus to better understand its role in the drug resistance mechanism.

## 1. Introduction

In the past decades, antibiotics have played a crucial role in fighting microbial infections. However, the overuse of antibiotics in clinical practice poses a serious risk to the public, and contributes to the emergence of multidrug-resistant (MDR) *Staphylococcus aureus*, which is responsible for various persistent and chronic infections [1]. Methicillin-resistant *S. epidermidis* (MRSE) is classified as a pathogen related to nosocomial and community-acquired infections [2]. *S. epidermidis* can become methicillin-resistant via the acquisition of the *mecA* gene, which is transferred with the help of staphylococcal chromosomal cassette mec (SCCmec) [3]. The failure of antibiotic therapy may be linked to the accumulation of resistance-relevant genes, as well as the flexibility changes in bacterial phenotypes [4]. MDR staphylococci can survive in hostile environments by forming biofilms or switching into small colony variants (SCVs) [1]. The genetic plasticity of *Staphylococcal* species has aided in the emergence of several drug-resistant strains, posing a significant therapeutic problem [5]. High prevalence of MRSE has been documented among clinical isolates in countries such as Iran (73.9 95%) [6], India (68%) [6], and Spain (37.2%) [7].

Coagulase-negative staphylococci (CoNS) are one of the most important human skin microbiota [8]. CoNS are also thought to be a significant reservoir of resistance genes. Recently, researchers have revealed that CoNS isolated from clinical samples and healthcare workers’ skin have a high rate of drug resistance and a variety of SCCmec types [9]. According to Bekoe et al. [10], tetracycline (63%) and ciprofloxacin (54%) were the drugs with the highest resistance identified in CoNS isolated from 401 urine samples of healthy individuals from Ghana. Due to the increase in drug resistance and invasive surgical procedures, CoNS are a global public health problem [11]. Even though CoNS are normally thought to be harmless, they are commonly linked to nosocomial illnesses, sparking increased interest in them as pathogens rather than as contaminants. The most common infections caused by CoNS are urinary tract infections (UTIs), eye infections, surgical site infections, and prosthetic joint infections [12]. *S. epidermidis* is the most common bacterium that is responsible for a significant number of nosocomial infections associated with medical implants (e.g., prosthetic devices and catheters), due to its ability to form a biofilm, which aids in attachment to inert biomaterial surfaces and increases antibiotic tolerance [12]. *S. epidermidis* infections are frequently chronic, with few distinguishing symptoms, so its distinct identification from other *Staphylococci* is necessary for accurate diagnosis [13]. In recent years, CoNS have attracted a lot of attention as pathogenic causes of infections in humans—particularly in immunocompromised, critically ill, or long-term hospitalized patients, and those with invasive medical devices, such as catheters. They have been connected to infections such as urinary tract infections, bloodstream infections, and infections caused by intrusive devices [14]. While distinguishing between harmless and pathogenic CoNS remains challenging, breakthroughs in diagnostic methods have increased our understanding of pathogenicity’s molecular mechanisms.

The *staphylococcal* cassette chromosome (SCC) is considered to be one of the most important mobile genetic elements in staphylococcal species, responsible for the mobility of methicillin resistance genes among different staphylococcal species [15]. To date, 14 variants of SCCmec types have been identified around the world, and these elements have been used in staphylococci evolution research, in addition to being used as molecular epidemiology tools in healthcare settings [16]. Among MRSE species, the IV(2B&5) SCCmec was reported as the most dominant SCCmec element type, and the SCCmec type III(3A) has been previously documented predominantly in healthcare-associated methicillin-resistant *Staphylococcus aureus* (MRSA) infections [17]. Although *S. epidermidis* is becoming an important opportunistic pathogen causing nosocomial infections, and tends to be more multidrug-resistant [18], it is often wrongly identified as a contaminant, despite causing many serious infections [14]. This study aimed to investigate and characterize the SCCmec elements, virulence factors, and ARGs in methicillin-resistant *S. epidermidis* strains isolated from clinical specimens.

## 2. Results

### 2.1. Isolation and Susceptibility Testing of S. epidermidis

The isolates were obtained from patients attending Soba University Hospital in Khartoum State suffering from chronic urinary tract infections (9SE) and chronic skin infections (14SP), and 5-day-old neonates suffering from sepsis (30SP) (Supplementary File S1, Table S1). The isolates were multidrug-resistant, and exhibited different levels of resistance to ciprofloxacin, tetracycline, cefoxitin, erythromycin, clindamycin, trimethoprim–sulfamethoxazole, gentamicin, chloramphenicol, and ampicillin. The isolates were resistant to cefoxitin, and they showed high levels of resistance to chloramphenicol and ampicillin. The isolates’ MIC values for chloramphenicol were reported to be ≥256 µgm/L, while the chloramphenicol (30 µg/L) disk diffusion susceptibility test showed no zone of inhibition with isolates 9SE and 30SP. A high level of resistance to ampicillin was observed in isolates 9SE and 14SP, in which the MIC reached 256 and 1024 µgm/L, respectively. Meanwhile, isolates 14SP and 30SP exhibited a small zone of inhibition (≤12), and an MIC value of 256 µg/L was recorded (Table 1).

### 2.2. Phenotypic Detection of Biofilm

By using the semi-quantitative microtiter plate method for estimation of the slime production, isolates 14SP and 30SP showed very weak positive results, with optical densities of 0.119 ± 0.04 and 0.116 ± 0.02, respectively. The isolate 9SE showed a weak positive result, with an OD of 0.318 ± 0.05. When using Congo red agar, isolates 14SP and 30SP produced dark pink colonies that were considered moderate slime producers, while isolate 9SE produced dark black colonies, which were considered positive (Supplementary File S1, Figure S1).

### 2.3. Characterization and Typing of Bacterial Genomes

The isolates (9SE, 14SP, and 30SP) were assembled in 2.5, 2.52, and 2.46 Mb, respectively; the number of contigs was ≤45, and the coverage was ≥370. The assembled genomes were identified at the species level using the PubMLST database, in which the isolates showed 100% identity with *S. epidermidis*. The isolates 9SE and 30SP were identified with sequence types (STs) 2 and 369, respectively, while the isolate 14SP was identified with novel alleles, and was assigned with a novel ID (736) by the Institut Pasteur team for the curation and maintenance of BIGSdb-Pasteur databases.

Comparing the genomics of our isolates to different strains of *S. epidermidis* (i.e., 949_S8, BPH0662, RP62A, and ATCC_12228) revealed that the species formed 3092 gene clusters, 2936 orthologous clusters, and 156 single-copy gene clusters. The isolates (9SE, 14SP, and 30SP) formed 96, 99, and 73 singleton proteins, respectively (Table 2) (Supplementary File S1, Figure S2). Comparison of the isolates’ whole genomic data with the BIGSdb-Pasteur database loci revealed the presence of the multidrug efflux pump (*QacA*) gene, which causes resistance to chlorhexidine, in isolate 9SE, while isolates 14SP and 30SP lacked this gene. The phenol-soluble modulins’ virulence factors (*PSM-b1* and *PSM-mec*), genes associated with biofilm formation (*icaA*, *icaB*, *icaC*, and *icaD*), and their regulator (*icaR*) were present in the 9SE isolate, while the formate dehydrogenase (*fdh*) gene was detected only in the 14SP and 30SP isolates (Table 3). The 9SE isolate was identified with a cluster of phage proteins, *Staphylococcal* nuclease family proteins, mobile element proteins, transposases, and *mecA* adaptor proteins (Supplementary File S1, Figure S3).

### 2.4. Staphylococcal Cassette Chromosome (SCC) Detection and Typing

**Figure 1:**
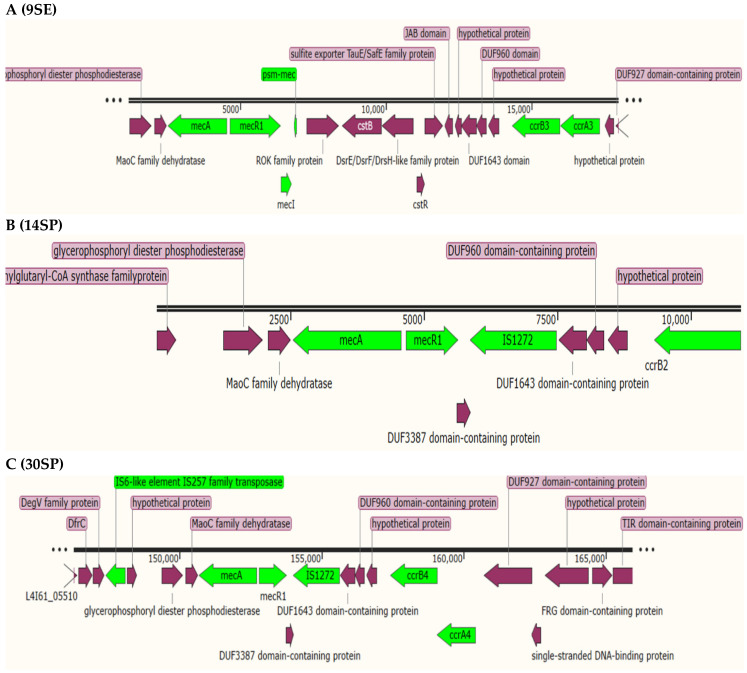
(**A**–**C**) Schematic representation of the SCC*mec* complex (green) in *S. epidermidis* isolates. The SCC*mec* is composed of the methicillin resistance gene (*mecA*), the *mecA* regulators (*mecI* and *mecR1*), and genes associated with integration and excision (ccr gene complex and IS) of the *mec*-gene complex.

**Figure 2:**
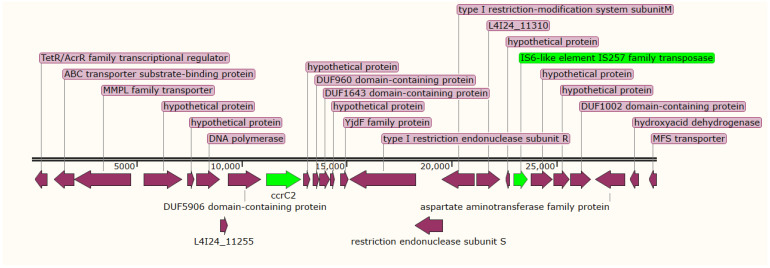
Schematic representation of the unique SCC in the 9SE strain, which contains ccrC2 and IS257, shown in green. The purple arrows showing other genes present in the same contig.

We used SCC*mec*Finder v1.2 to understand the diversity of mecA-encoding genes. According to the nomenclature used for MRSA, we identified a cassette of *ccrB3*, *ccrA3*, *mecA*, *mecR1*, and *mecI* genes corresponding to SCCmec type III(3A) in isolate 9SE, in which high nucleotide sequence identity (100%) was observed with *S. aureus* SCCmec type III(3A) (AB037671.1), and the *psm-mec* gene was found to be integrated into the SCCmec type III(3A) (Figure 1A). Additionally, 9SE contained another SCC cassette composed only of *ccrC2* and IS257 transposases (Figure 2). The 9SE *ccrA4* exhibited a high nucleotide similarity (99.85%) to *ccrA4* of *S. aureus* strain HDE288, while *ccrB4* exhibited a 94% nucleotide similarity to the *S. aureus* strain BK20781 *ccrB4* gene (Table 4).

Isolate 14SP contains a type IV(2B&5) SCCmec cassette that contains *mecA*, *dmecR1*, IS1272, *ccrB2*, *ccrC1* and *ccrA2*. The *ccrB4* gene exhibited 100% identity to *S. aureus* strain CHE482′s cassette chromosome recombinase A (*ccrA4-2*) (EF126186.1) (Figure 1B). Prediction of the SCCmec cassette of the 30SP strain revealed the presence of SCCmec type VI(4B), which showed 87.47% coverage of the *S. aureus* strain HDE288 type VI SCCmec element, with a cassette consisting of the type B1 *mec* complex (IS257-*mecA-mecR*-IS1272) and the ccrA/B (type IV) (Figure 1C).

### 2.5. Antimicrobial Resistance Genes, Mobile Genetic Elements, and Virulence Factors

We investigated the genomic data of *S. epidermidis* for the presence of specific genetic features associated with bacterial pathogenicity and drug resistance. A total of 11 ARGs was identified as chromosomally mediated or present in plasmids; these genes encode for proteins causing decreased susceptibility to methicillin (*mecA*), penicillin (*blaZ*), fusidic acid (*fusB*), fosfomycin (*fosB*), tetracycline (*tet*(*K*)), aminoglycosides (*aadD*, aac(6′)-aph(2″)), fluoroquinolone (MFS antibiotic efflux pump), trimethoprim (*dfrG*), macrolide (*msr*(*A*)), and chlorhexidine (*qacA*) (Table 5). Isolate 9SE was identified as having seven ARGs (i.e., *fosB*, *fusB*, *mecA*, *aac*(*6′*)*-aph*(*2*″), *fosD*, *qacA*, and *mupA*). Genes encoding for fosfomycin (*fosB*), antiseptic resistance protein (*qacA*), and type II restriction enzymes (DpnII) were identified as being clustered in the 9SE plasmid, flanked by replication initiation protein A (*repA*) (Supplementary File S1, Figure S4). Isolate 14SP harbored different ARGs (i.e., *aadD*, *msr*(A), *fosB*, *blaZ*, *dfrG*, *fusB*, *mecA*, and *aac*(*6′*)*-aph*(*2*″)) and/or a point mutation (T173A) in *S. aureus gyrB*, conferring resistance to aminocoumarin.

As shown in Table 5, different ARGs causing resistance to various antibiotics used for the treatment of staphylococcal infections were noted in the 30SP strain. Class A beta-lactamase gene (*blaZ*), macrolide resistance gene (*msr*(*A*), phage protein, and replication initiation protein A (*repA*) were identified as being clustered in the plasmid (*p*SER10C-2) of 30SP (Supplementary File S1, Figure S5). The (MFS) antibiotic efflux pump (*tet*(*K*)) was identified in the 30SP isolate, with 100% identity and coverage; this gene was detected to be flanked by replication initiation protein (*repA*) and topoisomerase. Moreover, the MFS antibiotic efflux pump gene (*norA*) was identified in 9SE, 14SP, and 30SP, with identity of 100%, 98.7%, and 99.5%, respectively.

Genes responsible for adherence—including autolysin (*atl*), cell-wall-associated fibronectin-binding protein (*ebh*), elastin-binding protein (*ebp*), and Ser–Asp-rich fibrinogen-binding proteins (*sdrG*)—were identified in the three isolates (9SE, 14SP, and 30SP) and the control (RP62A). Similarly, the cysteine protease (*sspB*), lipases (*geh* and *lip*), serine V8 protease (*sspA*), thermonuclease (*nuc*), and beta-hemolysin (*hlb*) genes were detected in all four isolates, including the reference, while intercellular adhesin genes (i.e., *icaA*, *icaB*, *icaC*, *icaD*, and *icaR*) and Ser–Asp-rich fibrinogen-binding proteins (*sdrF*) were documented only in the 9SE strain (Supplementary File S1, Table S2).

### 2.6. Phylogenetic Analysis

**Figure 3:**
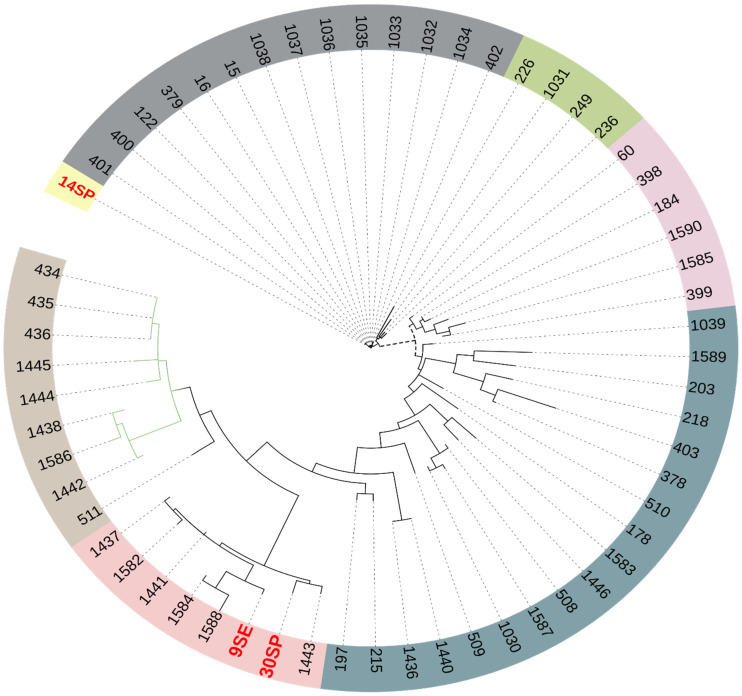
Circular representation of the maximum likelihood phylogenetic tree of *S. epidermidis*, including 9SE, 14SP, and 30SP in red, and the most related strain (57) of *S. epidermidis* submitted from Africa in the PubMLST database. Different clades shown in different colors.

In order to see the relationships between *S. epidermidis* strains, SNP-based phylogenetic analysis was performed after the alignment of the core genome. Isolate 14SP was located in a separate clade, while the 9SE and 30SP isolates were clustered in a clade containing *S. epidermidis* strains from different African countries—including Nigeria (1441, 1437, and 1443) and Ghana (1584 and 1582)—that were isolated from patients with bacteremia (Figure 3); the metadata of the reference strains used for comparison can be found in Supplementary File S2.

## 3. Discussion

*S. epidermidis* is part of the normal human flora, predominantly colonizing the adult skin [20], and on many occasions it is wrongly identified as a contaminant, despite causing many serious infections [14]. In recent years, it has become an important opportunistic pathogen causing nosocomial infections, and it tends to be multidrug-resistant [18]. In this study, three clinical isolates of *S. epidermidis* were identified as multidrug-resistant, showing resistance to various antibiotics, including cefoxitin. The staphylococcal cassette chromosome (SCC) is considered to be one of the most important mobile genetic elements in staphylococci, being responsible for the mobility of methicillin resistance genes among different staphylococcal species [15]. Since the first reports of SCCmec I, II, and III in the early 2000s, up to 14 variants of SCCmec elements have been reported by various researchers around the world, and these elements have been used for the study of staphylococci’s evolution, and also used as molecular epidemiology tools in healthcare settings [16]. These investigations have also raised concerns about the presence of SCCmec in clinical isolates being misinterpreted. In this study, for the first time in Sudan, we reported the presence of the SCCmec type among MRSE; a cassette of *ccrB3*, *ccrA3*, *mecA*, *mecR1*, and *mecI* genes corresponding to SCCmec type III(3A) was identified in isolate 9SE, with high nucleotide sequence identity (100%) with *S. aureus* SCCmec type III(3A) (AB037671.1). The SCCmec type III(3A) has been previously documented predominantly in healthcare-associated MRSA infections [17], which could indicate the possible horizontal transmission of this cassette from *S. aureus* to *S. epidermidis* species. The IV(2B&5) SCCmec was reported as the most dominant SCCmec element among methicillin-resistant *S. epidermidis* (MRSE) [17]; here, it was documented in isolate 14SP, which contains the *mecA*, *dmecR1*, *IS1272*, *ccrB2*, *ccrC1*, and *ccrA2* genes. As reported in this study, the combination of *ccr1*, *ccr2*, and *ccrC* is usually detected in the IV(2B&5) SCCmec type [17]. In addition to the presence of the *ccrA/B* element in *i*solate 9SE, an extra *ccrB4* was uniquely identified in this study; this new composite element of *ccr* elements needs more focus to better understand its role in the drug resistance mechanism. The existence of SCCmec type VI(4B) was discovered in the 30SP strain, which demonstrated 87.47% identity with the *S. aureus* strain HDE288 type-VI SCCmec element. The cassette consists of the type B1 *mec* complex (IS257-mecA-mecR-IS1272) and *ccrAB* (type 4) genes. This is consistent with a previous study in which the authors reported the presence of SCCmec type VI(4B) in MRSE [21].

Bacterial virulence is a complex topic that requires research from a clinical, molecular, and genetic perspective. In clinical terminology, virulence refers to a pathogen’s inherent ability to induce specific clinical symptoms that are thought to be linked to the production of different virulence factors [11]. The virulence and pathogenicity of three clinical *S. epidermidis* strains were investigated in this study. Even though strains belonging to the clonal type ST2 have been over-represented—especially in *S. epidermidis* newborn sepsis—none of the virulence factors could be used as a reliable factor for identification of a specific strain as harmful or commensal. 

The insertion sequence IS256 and the *ica* genes are usually found in ST2 clonal types, which have been linked to biofilm generation and hospital-acquired infection [22]. This is consistent with our study, in which the ST2 strain was found in a hospitalized patient suffering from a chronic urinary tract infection. Multiple virulence factors have been discovered in the *S. aureus* genome, according to numerous studies; *S. epidermidis* carried nearly half of those factors [23]. The 9SE MRSE-ST2 strain was reported with the fusidic acid resistance gene (*fusB*); the clinical clones of MRSE-ST2 became globally epidemic, resulting in outbreaks [24]; and the fusidic acid MRSE-ST2 was the most common ST among clinical isolates in the USA, German, China, and others [25,26,27]. The circulation of such strains in hospitals was reported in South Africa, as shown in phylogenetic tree strains 1033, 1035, 1036, and 1037, which belonged to ST2, and were isolated from the blood of hospitalized patients in Pretoria. This was consistent with our findings, where 9SE was recovered from a hospitalized patient suffering from chronic UTI, indicating that the MRSE-ST2 clone has disseminated in our region.

Prophages, insertion sequences, and SCCmec-like cassettes were among the mobile genetic elements discovered in *S. epidermidis* genomes. Other possible virulence factors include proteases (serine and cysteine proteases), lipases, and hemolysin (e.g., beta/delta hemolysin) loci. The existence of mobile genetic elements and virulence factors could promote horizontal gene transfer between staphylococci and increase their pathogenicity [22]. The present study noted that seven ARGs (i.e., *fosB*, *fusB*, *mecA*, *aac*(*6′*)*-aph*(*2″*), *fosD*, *qacA*, and *mupA*) were present in isolate 9SE. Different ARGs (*aadD*, *msr*(*A*), *fosB*, *blaZ*, *dfrG*, *fusB*, *mecA*, and *aac*(*6′*)*-aph*(*2″*) were found in isolate 14SP, as well as a point mutation (T173A) in the *gyrB* gene that causes resistance to aminocoumarin. Moreover, the multidrug efflux pump (*QacA*) gene was observed in isolate 9SE, causing resistance to chlorhexidine, which is used for skin disinfection before surgeries [28]. In the hospital setting, chlorhexidine is the most frequently used antiseptic [29]; the presence of isolates resistant to chlorhexidine and the most commonly used antibiotics represents a serious public health problem. The presence of these ARGs was inconsistent with phenotypic antimicrobial resistance findings. We noted that all of the isolates showed varying levels of resistance to ampicillin, which could be attributed to the presence of the class A beta-lactamase (*blaZ*) gene and methicillin resistance mechanism [30]. The high level of resistance to methicillin could be attributed to widely prescribed β-lactam antibiotics for the treatment of different bacterial infections in Sudan [31], which are commonly used for prophylaxis before surgeries [28]. In the 30SP strain, we detected different ARGs that may induce resistance to conventional drugs used to treat staphylococcal infections. The presence of these genes is reflected in phenotypic resistance to ciprofloxacin, tetracycline, cefoxitin, trimethoprim–sulfamethoxazole, gentamicin, and ampicillin. The plasmid (*p*SER10C-2) of 30SP contained the genes related to resistance for class A beta-lactamase (*blaZ*), macrolide resistance (*msr*(*A*)) gene, phage protein, and replication initiation protein A (repA). Phage protein and replication initiation protein A (*repA*) are frequently found in multidrug-resistant plasmids [32]. Researchers have noted that most staphylococcal multidrug-resistant plasmids possess a highly conserved repA protein [33]. The major facilitator superfamily (MFS) antibiotic efflux pump (*tet*(*K*)) gene was found to be flanked by replication initiation protein and topoisomerase in the 30SP isolate. This could be the reason behind the high resistance rate against tetracycline observed in the 30SP isolate. Despite the absence of the chloramphenicol resistance gene (*cat*) in our isolates, they exhibited a high level of resistance (MIC ≥ 256) to chloramphenicol, which could be attributed to the presence of other resistance mechanisms such as multidrug efflux pumps in our isolates [34]. A high level of resistance (MIC = 256) was observed in isolates 14SP and 30SP, which could be attributed to the presence of the MFS antibiotic efflux pump, which is associated with the fluoroquinolone resistance mechanism [35].

*S. epidermidis* has an open-pan genome that includes core genes (80%) and variable genes (20%) [36]. The pan-genome analysis of the isolates revealed the presence of phenol-soluble modulin (PSM) genes in isolate 9SE; the PSM genes have recently been identified as significant virulence factors, most notably in aggressive strains of *S. aureus* [37]. The presence of the IS256 and *ica* genes was used as a measure of the pathogenicity of *S. epidermidis* [38]. The 9SE strain possessed genes associated with biofilm formation (*icaA*, icaB, icaC, and *icaD*), IS256, and the gene regulator (*icaR*); biofilm-forming isolates have more capacity to adhere to medical devices, thus increasing the risk of device-associated infections [20]. Isolates harboring the biofilm-associated genes, the antiseptic resistance gene *qacA*, the cassette genes (*ccrA* and *ccrB*), and the IS256-like transposase gene were more likely to be found in patients who had several procedures as a consequence of therapeutic failure [39]. Although strains 14SP and 30SP lack the IS256 and *ica* genes, they were positive for other adhesins such as autolysin (*atl*), cell-wall-associated fibronectin-binding protein (*ebh*), elastin-binding protein (*ebp*), and Ser–Asp-rich fibrinogen-binding proteins (*sdrG*). Similarly, they were positive for other virulence factors, including cysteine protease (*sspB*), lipases (*geh* and *lip*), serine V8 protease (*sspA*), thermonuclease (*nuc*), and beta-hemolysin (*hlb*), which could increase their pathogenicity and differentiate them from the commensal strains [38].

Phenotypic detection of biofilm formation showed that all of the isolates are weak biofilm producers; isolate 9SE had higher OD values than 14SP and 30SP, which could be attributed to the presence of *ica* genes. Although isolates 14SP and 30SP are *ica-*gene-negative isolates they showed weak phenotypic biofilm production, which could be attributed to the presence of *ica*-independent mechanisms of biofilm production [40].

## 4. Methods

### 4.1. Bacterial Isolates

Three isolates of coagulase-negative staphylococci (CoNS) species were identified as a part of a study conducted in Soba University Hospital to investigate the presence of drug-resistant bacteria in clinical settings during the period between November and May of 2021. 

The clinical samples were processed as a part of the daily routine of clinical sample identification and processing at Soba University Hospital. The isolates were obtained from different patients suffering from chronic urinary tract infections (9SE) and chronic skin infections (14SP), as well as 5-day-old neonates suffering from sepsis (30SP) (Supplementary File S1, Table S1), and were selected based on their resistance to cefoxitin. The clinical isolates were identified by standard biochemical tests [41], and according to their colors and growth characteristics on chromogenic media. 

### 4.2. Antimicrobial Susceptibility Testing

The isolates were subjected to disk diffusion antimicrobial susceptibility testing using the following antibiotics: ciprofloxacin (5 µg), tetracycline (30 µg), cefoxitin (30 µg), gentamicin (10 µg) erythromycin (15 µg), clindamycin (2 µg), trimethoprim–sulfamethoxazole (25 µg), and chloramphenicol (30 µg). The minimum inhibitory concentrations (MICs) of ciprofloxacin, tetracycline, gentamicin, chloramphenicol, and ampicillin were determined using the microtiter broth dilution method [42]. Overnight bacterial growth was adjusted to 5-10^5^ CFU/mL in Mueller–Hinton (MH) broth and then used for the preparation of twofold serial dilution of antibiotics; then, 100 μL of broth was poured into each well. The antibiotic concentrations used were in the range of 2 to 1024 μg/mL [43]. MIC results were interpreted according to the CLSI guidelines [19].

The American Type Culture Collection (ATCC) S. *aureus* (ATCC 25923) strain was used for quality control of media and antibiotic disks. Results were interpreted according to the Clinical and Laboratory Standards Institute (CLSI) guidelines [19]. 

### 4.3. Phenotypic Detection of Biofilm Production

Phenotypic biofilm assay was conducted using two methods: the microtiter plate (MtP) and Congo red agar (CRA) methods, as described by Chaieb et al. [44]. In the MtP method, the isolates were cultured overnight at 37 °C in brain–heart infusion broth (BHI, HiMedia, Mumbai, India) with 2% (*w*/*v*) glucose, and then the culture was diluted to 1: 100, and a total of 200 µL of diluted cultures was transferred to a 96-well polystyrene microplate (Nunc, Denmark), where each sample was tested in quintuplicate, including the positive control (*S. epidermidis*, CIP106510). After overnight incubation at 37 °C, the wells were washed, air-dried, fixed with 95% ethanol, and stained with 1% crystal violet for 5 min. The wells were then washed three times with distilled water, air-dried, and the optical density (OD) of each well was measured at 570 nm using an ELISA plate reader. OD of less than 0.1 was considered negative, OD values in the range of 0.126 and 0.9 were considered weakly positive, and OD ≥ 1 was considered strongly positive [45].

The CRA method was used to estimate the slime production according to the change in the color of grown bacteria on CRA. BHI agar supplemented with sucrose (36 g L^−1^) and Congo red (0.8 g L^−1^) was prepared for inoculation of *S. epidermidis* strains, and then incubated overnight at 37 °C. Deep black colonies with a metallic sheen were considered positive, dark pink colonies were considered moderate slime producers, and light pink colonies were considered negative [45].

### 4.4. Whole-Genome Sequencing (WGS) and Data Analysis

Bacterial genomic DNA was extracted from overnight growth via the quinidine chloride protocol [46], and then the quality of extracted DNA was estimated using NanoDrop and Qubit (Thermo Scientific, Waltham, MA, USA). Paired-end reads (2 × 150 bp) were generated from WGS using Illumina HiSeq 2500 (Novogene, Beijing, China). Before the genomic data analysis, the reads with low-quality, adaptors and reads less than 200 bp were removed using Trimmomatic 0.36 [47]. The PATRIC server was used for de novo genome assembly, and then the assembled reads were submitted to the MLST 2.0 and PubMLST [48] databases for species identification. Subspecies identification and curation of novel strains was achieved with the help of the Institut Pasteur team for the curation and maintenance of the BIGSdb-Pasteur databases. Genome annotation was achieved with the PATRIC server and NCBI Prokaryotic Genome Annotation Pipeline (PGAP) [49]. 

### 4.5. Prediction of Resistome and Mobilome

Resistance Gene Identifier (RGI) and ResFinder [50] were used for the prediction of antimicrobial resistance genes (ARGs). Genes were identified based on a >80% hit length and >90% sequence identity. SCCmecFinder v.1.2 [51] was used for the identification of *mecA*-carrying SCC*mec*; the default parameters were applied with minimum thresholds of sequence identity (>90%) and sequence coverage (>60%). Plasmids were screened using the plasmidSPAdes tool v3.15.4 [52] and PlasmidFinder 2.1 (Center for Genomic Epidemiology, DTU, Lyngby, Denmark). SnapGene Viewer v.6.0.2 was used for the visualization of gene cassettes. Virulence genes were investigated using the virulence factor database (VFDB) [53], using *S. epidermidis* RP62A as a reference strain for comparison [54].

### 4.6. Pan-Genome Analysis and Phylogenetic Tree

The rapid annotation of prokaryotic genomes (Prokka) [55] tool was used for annotation of the assembled contigs of our isolates and reference strains of *S. epidermidis* (949_S8, BPH0662, RP62A, and ATCC_12228). Roary [56], the pan-genome pipeline, was used for quick generation of a core gene alignment from the gff3 files generated from Prokka, adjusted with identity for BLASTp (95%) and 99% for isolates’ genes to be considered a core. The Gene Presence tool of the BIGSdb-Pasteur databases was used to compare whole-genome data of the isolates with the database-defined loci of an annotated genome used for comparison.

Maximum-likelihood-based inference of large phylogenetic trees (Galaxy Version 8.2.4 + galaxy2) was generated by the phylogenetic reconstruction tool RAxML, and the alignment of the core genes generated by Roary was used as the input, while GTRGAMMA was used as a substitution model.

Another phylogenetic tree was generated against all genomes (57) of *S. epidermidis* submitted from Africa in the PubMLST database. The Interactive Tree of Life (iTOL) v5 was used for the visualization of the tree.

## 5. Limitations

Our work had some limitations that should be mentioned. MICs were determined for a limited number of antibiotics due to the unavailability of others in our region, and we faced some difficulties in importing them from abroad.

## 6. Conclusions

This study focused on the genomic characteristics of methicillin-resistant *S. epidermidis*, in which we detected novel MDR strains circulating in our hospital setting; these isolates were methicillin-resistant, and carried different types of staphylococcal cassette chromosome (SCC) elements and insertion sequences (ISs) associated with integration and precise excision of the mec-gene complex. Isolate 9SE was identified as having the chlorhexidine resistance (*qacA*) gene; chlorhexidine is the most commonly used antiseptic, which represents a serious public health problem. Additionally, the 9SE strain possessed different genes associated with biofilm formation (*icaA*, *icaB*, *icaC*, and *icaD*), which could increase the risk of device-associated hospital-acquired infections. The 9SE strain was identified with extra ccrB4, which was unique to this study; this new composite element of the *ccr* type needs more focus to better understand its role in the drug resistance mechanism.

## Figures and Tables

**Table 1:** Antimicrobial susceptibility testing of selected antimicrobial agents used against bacterial isolates.

Antibiotic	*Staphylococcus epidermidis*					
	9SE		14SP		30SP	
	MIC (μg/mL)	Disk Diffusion Susceptibility ^a^	MIC (μg/mL)	Disk Diffusion Susceptibility ^a^	MIC (μg/mL)	Disk Diffusion Susceptibility ^a^
Ciprofloxacin	≤2	S	256	R	256	R
Tetracycline	32	R	≤2	S	32	R
Cefoxitin	-	R	-	R	-	R
Erythromycin	-	S	-	R	-	S
Clindamycin	-	R	-	S	-	S
Trimethoprim–sulfamethoxazole	-	S	-	R	-	R
Gentamicin	≤2	S	≤2	S	≥1024	R
Chloramphenicol	512	R	256	R	512	R
Ampicillin	256	R	≥1024	R	4	R

Abbreviations: R = resistant, S = susceptible, - = not tested, mm = millimeter. ^a^ Antimicrobial susceptibility testing determined according to CLSI guidelines [19].

**Table 2:** Summary of orthologous clusters and single-copy gene clusters of isolated reference strains of *S. epidermidis.*

Strains	Proteins	Clusters	Singletons
*S. epidermidis* 9SE	2285	2184	96
*S. epidermidis* 14SP	2321	2212	99
*S. epidermidis* 30SP	2230	2150	73
*S. epidermidis* 949_S8	2119	2094	23
*S. epidermidis* BPH0662	2654	2412	182
*S. epidermidis* RP62A	2401	2304	60
*S. epidermidis* ATCC_12228	10,252	2583	2766

**Table 3:** Presence of genes among *S. epidermidis* isolates (9SE, 14SP, and 30SP) and reference strains (ATCC12228, RP62A949_S8, and BPH0662) used for comparison.

S. epidermidis ID	Acetyltransferase	CcrA	CcrB	Fdh	IcaA	IcaB	IcaC	IcaD	IcaR	IS256-like	mecA	mecC	PSM-b1	PSM-mec	QacA	Tn554
ATCC12228	0	1	1	0	0	0	0	0	0	0	0	0	1	0	0	0
RP62A	1	1	1	0	1	1	1	1	1	1	1	1	1	1	0	1
949_S8	0	0	0	0	0	0	0	0	0	0	0	0	1	0	0	0
BPH0662	1	1	1	0	1	1	1	1	1	1	1	1	1	1	0	1
9SE	1	1	1	0	1	1	1	1	1	1	1	1	1	1	1	0
14SP	1	1	1	1	0	0	0	0	0	1	1	1	0	0	0	0
30SP	0	0	1	1	0	0	0	0	0	0	1	1	0	0	0	0

1 = Gene present, 0 = gene absent.

**Table 4:** SCCmec complex types and their positions in *S. epidermidis*.

ID	SCCmec Genes	Type/Temp Coverage	Contig	Identity	Position in Contig
9SE	ccrC2-allele-1:1:KR187111	III(3A)/63.85% XIII(9A)	20	96.44	11,178..12,863
	ccrB3:1:852082:AB037671		21	100.00	14,339..15,967
	ccrA3:1:852082:AB037671		21	100.00	15,988..17,334
	mecA:12:AB505628		21	100.00	2522..4531
	mecR1:1:D86934		21	100.00	4638..6395
	mecI:1:D86934		21	100.00	6395..6766
14SP	mecA:12:AB505628	IV(2B&5)/74.73%	25	100.00	2560..4569
	dmecR1:1:AB033763		25	100.00	4666..5652
	IS1272:3:AM292304		25	100.00	5641..7483
	ccrB4:2:BK20781:FJ670542		15	92.20	7117..8745
	ccrC1-allele-7:1:EF190468		11	100.00	74,999..76,675
	subtype-IVc(2B):3:81108:AB096217		11	100.00	85,144..86,298
	ccrA2:7:81108:AB096217		11	100.00	90,315..91,664
	ccrB2:7:81108:AB096217		25	100.00	9325..10,914
30SP	mecA:12:AB505628	VI(4B)/87.47%	3	100.00	150,689..152,698
	dmecR1:1:AB033763		3	100.00	152,795..153,781
	IS1272:3:AM292304		3	100.00	153,770..155,612
	ccrB4:2:BK20781:FJ670542		3	94.05	157,433..159,061
	ccrA4:1:HDE288:AF411935		3	99.85	159,058..160,419
	IS1272:2:AB033763		1	91.12	213,133..214,709

Note: IS257 is also known as IS431.

**Table 5:** Antimicrobial resistance genes present in *S. epidermidis* isolates.

*S. epidermidis*	Contig	ARGs	Position	Coverage	Identity
9SE	5	*fosB*	78015-78443	100%	99.5%
	35	*fusB*	1634-993	100%	100%
	21	*mecA*	2522-4528	100%	100%
	36	*aac*(*6′*)-*aph*(*2″*)	1519-80	100%	99.9%
	22	*fosD*	78015-78443	100%	99.5%
		*qacA*	12368-13912	100%	100%
	31	mupA	158-3232	100%	99.96%
14SP	28	*aadD*	191-952	100%	100%
	11	*msr*(*A*)	1716-250	100%	99.7 %
	7	*fosB*	71675-72103	100%	96.5%
		*blaZ*	130038-130883	100%	100%
	6	*dfrG*	58760-59257	100%	100%
	18	*fusB*	45606-46247	100%	100%
	25	*mecA*	2560-4566	100%	100%
	34	*aac*(*6′*)-*aph*(*2″*)	1839-400	100%	100%
30SP	2	*fosB*	225233-225661	100%	96.27%
	15	*msr*(*A*)	13445-14911	100%	99.72%
		*blaZ*	8566-9411	100%	99.88%
	3	*mecA*	150689-152695	100%	100%
		*fusC*	167439-168077	100%	99.21%
	18	*tet*(*K*)	1122-2501	100%	100%

## Data Availability

The data of this project were submitted to GenBank under the Bioproject PRJNA767482, Accession numbers JAKKEA000000000 (9SE), JAKKED000000000 (14SP), and JAKKEK000000000 (30SP), and under the following biosamples: SAMN25227152 (9SE), SAMN25227155 (14SP), and SAMN25227162 (30SP). Additional tables and figures are listed in the Supplementary Materials.

## Supplementary Materials

The following supporting information can be downloaded at: https://www.mdpi.com/article/10.3390/antibiotics11070861/s1, Supplementary File S1, Figure S1: Congo red agar; Supplementary File S1, Figure S2: S. epidermidis variable genome; Supplementary File S1, Figure S3: Linear representation of S. epidermidis (9SE) phage proteins, Staphylococcal nuclease family protein, mobile element proteins, transposases, and MecA adaptor protein; Supplementary File S1, Figure S4: Circular representation of clustered ARGs in isolate 9SE, the repA gene; Supplementary File S1, Figure S5: Circular representation of S. epidermidis (30SP) plasmid pSER10C-2; Supplementary File S1, Table S1: Genomic characteristics of isolated organisms; Supplementary File S1, Table S2: The virulence factors identified in the isolates; Supplementary File S2 contained the metadata for isolates used in the phylogenetic tree.
