# Supplementary material for: Whole-Genome Sequence of Multidrug-Resistant Methicillin-Resistant Staphylococcus epidermidis Carrying Biofilm-Associated Genes and a Unique Composite of SCCmec
Source: Antibiotics (Basel). 2022 Jun 24;11(7):861. doi: 10.3390/antibiotics11070861 (PMC9312184; doi:10.3390/antibiotics11070861)
Supplement: Supplementary file 1 [file antibiotics-11-00861-s001.zip › Supplementary file S1.pdf]

Table S1. Genomic characteristics of isolated organisms

ID	Isolation source	Age	Gender	PubMLST	Identity	ST	Biosample	Source	bp	Contigs	GC	CDS	tRNA	rRNA	Coverage	N50
9SE	Urinary tract infection	25	Male	*Staphylococcus epidermidis*	100%	2	SAMN25227152	Chronic UTI	2505680	45	32.11	2413	52	4	399	165065
14SP	Wound (chronic skin infection)	13	Female	*Staphylococcus epidermidis*	100%	Novel	SAMN25227155	Chronic skin infection	2522530	14	32.03	2422	52	3	370	133978
30SP	Blood (sepsis)	5 Days	Female	*Staphylococcus epidermidis*	100%	369	SAMN25227162	Sepsis	2469456	28	31.94	2344	52	4	471	208563

Table S2. The virulence factors identified in the isolates

Gene		RP62A	9SE	14SP	30SP
**Adherence (22)**					
Autolysin	*atl*	SERP0636	orf01170	orf00483	orf01589
Cell wall associated fibronectin binding protein	*ebh*	SERP1011	orf00357	orf00174	orf00358
Clumping factor A	*clfA*	-	-	-	-
Clumping factor B	*clfB*	-	-	-	-
Collagen adhesion	*cna*	-	-	-	-
Elastin binding protein	*ebp*	SERP1048	orf00394	orf00211	orf00321
Extracellular adherence protein/MHC analogous protein	*eap/map*	-	-	-	-

Fibrinogen binding protein	*efb*	-	-	-	-
Fibronectin binding proteins	*fnbA*	-	-	-	-
Fibronectin binding proteins	*fnbB*	-	-	-	-
Intercellular adhesin	*icaA*	SERP2293	orf01027	-	-
Intercellular adhesin	*icaB*	SERP2295	orf01029	-	-
Intercellular adhesin	*icaC*	SERP2296	orf01030	-	-
Intercellular adhesin	*icaD*	SERP2294	orf01031	-	-
Intercellular adhesin	*icaR*	SERP2292	orf01026	-	-
Ser-Asp rich fibrinogen-binding proteins	*sdrC*	-	-	-	-
Ser-Asp rich fibrinogen-binding proteins	*sdrD*	-	-	-	-
Ser-Asp rich fibrinogen-binding proteins	*sdrE*	-	-	-	-
Ser-Asp rich fibrinogen-binding proteins	*sdrF*	SERP0026*	orf00966	-	-
Ser-Asp rich fibrinogen-binding proteins	*sdrG*	SERP0207	orf00560	orf00806	orf01176
Ser-Asp rich fibrinogen-binding proteins	*sdrH*	SERP1487	orf01368	orf01385	-
Staphylococcal protein A	*spa*	-	-	-	-
**Enzyme (15 Items)**					
Cysteine protease	*sspB*	SERP2390	orf01117	orf01141	orf00918
Cysteine protease	*sspC*	-	-	-	-
Hyaluronate lyase	*hysA*	-	-	-	-

Lipase	*geh*	SERP2388	orf01116	orf01142	orf00917
Lipase	*lip*	SERP2297	orf01031	orf01231	orf00828
Serine V8 protease	*sspA*	SERP1397	orf02006	orf00698	orf00001
Serine protease	*splA*	-	-	-	-
Serine protease	*splB*	-	-	-	-
Serine protease	*splC*	-	-	-	-
Serine protease	*splD*	-	-	-	-
Serine protease	*splE*	-	-	-	-
Serine protease	*splF*	-	-	-	-
Staphylocoagulase	*coa*	-	-	-	-
Staphylokinase	*sak*	-	-	-	-
Thermonuclease	*nuc*	SERP0891	orf00240	orf00058	orf00475
**Immune evasion (5 Items)**					
AdsA	*adsA*	-	-	-	
CHIPS	*chp*	-	-	-	
Capsule	--	-	-	-	
SCIN	*scn*	-	-	-	
Sbi	*sbi*	-	-	-	
**Secretion system (12 Items)**					
Type VII secretion system	*esaA*	-	-	orf00896	orf00781

Type VII secretion system	*esaB*	-	-	-	orf00779
Type VII secretion system	*esaD*	-	-	orf00905	-
Type VII secretion system	*esaE*	-	-	orf00903	-
Type VII secretion system	*esaG*	-	-	orf00907	orf00762
Type VII secretion system	*essA*	-	-	orf01811	orf00763
Type VII secretion system	*essB*	-	-	orf00897	orf00765
Type VII secretion system	*essC*	-	-	orf00899	orf00780
Type VII secretion system	*esxA*	-	-	orf00900	orf00778
Type VII secretion system	*esxB*	-	-	orf00895	orf00777
Type VII secretion system	*esxC*	-	-	orf00902	orf00782
Type VII secretion system	*esxD*	-	-	orf00901	-
**Toxin (74 Items)**					
Alpha hemolysin	*hly/hla*	-	-	-	-
Beta hemolysin	*hlb*	SERP2544	orf01000	orf01906	orf01048
Delta hemolysin	*hld*	SERP1489	-	-	-
Enterotoxin A	*sea*	-	-	-	-
Enterotoxin B	*seb*	-	-	-	-
Enterotoxin C	*sec*	-	-	-	-
Enterotoxin D	*sed*	-	-	-	-

Enterotoxin E	*see*	-	-	-	-
Enterotoxin G	*seg*	-	-	-	-
Enterotoxin H	*seh*	-	-	-	-
Enterotoxin I	*sei*	-	-	-	-
Enterotoxin J	*sej*	-	-	-	-
Enterotoxin Yent1	*yent1*	-	-	-	-
Enterotoxin Yent2	*yent2*	-	-	-	-
Enterotoxin-like K	*selk*	-	-	-	-
Enterotoxin-like L	*sell*	-	-	-	-
Enterotoxin-like M	*selm*	-	-	-	-
Enterotoxin-like N	*seln*	-	-	-	-
Enterotoxin-like O	*selo*	-	-	-	-
Enterotoxin-like P	*selp*	-	-	-	-
Enterotoxin-like Q	*selq*	-	-	-	-
Enterotoxin-like R	*selr*	-	-	-	-
Enterotoxin-like U	*selu*	-	-	-	-
Exfoliative toxin type A	*eta*	-	-	-	-
Exfoliative toxin type B	*etb*	-	-	-	-
Exfoliative toxin type C	*etc*	-	-	-	-

Exfoliative toxin type D	*etd*	-	-	-	-
Exotoxin	*set10*	-	-	-	-
Exotoxin	*set11*	-	-	-	-
Exotoxin	*set12*	-	-	-	-
Exotoxin	*set13*	-	-	-	-
Exotoxin	*set14*	-	-	-	-
Exotoxin	*set15*	-	-	-	-
Exotoxin	*set16*	-	-	-	-
Exotoxin	*set17*	-	-	-	-
Exotoxin	*set18*	-	-	-	-
Exotoxin	*set19*	-	-	-	-
Exotoxin	*set1*	-	-	-	-
Exotoxin	*set20*	-	-	-	-
Exotoxin	*set21*	-	-	-	-
Exotoxin	*set22*	-	-	-	-
Exotoxin	*set23*	-	-	-	-
Exotoxin	*set24*	-	-	-	-
Exotoxin	*set25*	-	-	-	-
Exotoxin	*set26*	-	-	-	-

Exotoxin	*set2*	-	-	-	-
Exotoxin	*set30*	-	-	-	-
Exotoxin	*set31*	-	-	-	-
Exotoxin	*set32*	-	-	-	-
Exotoxin	*set33*	-	-	-	-
Exotoxin	*set34*	-	-	-	-
Exotoxin	*set35*	-	-	-	-
Exotoxin	*set36*	-	-	-	-
Exotoxin	*set37*	-	-	-	-
Exotoxin	*set38*	-	-	-	-
Exotoxin	*set39*	-	-	-	-
Exotoxin	*set3*	-	-	-	-
Exotoxin	*set40*	-	-	-	-
Exotoxin	*set4*	-	-	-	-
Exotoxin	*set5*	-	-	-	-
Exotoxin	*set6*	-	-	-	-
Exotoxin	*set7*	-	-	-	-
Exotoxin	*set8*	-	-	-	-
Exotoxin	*set9*	-	-	-	-

Gamma hemolysin	*hlgA*	-	-	-	-
Gamma hemolysin	*hlgB*	-	-	-	-
Gamma hemolysin	*hlgC*	-	-	-	-
Leukocidin M	*lukF-like*	-	-	-	-
Leukocidin M	*lukM*	-	-	-	-
Leukotoxin D	*lukD*	-	-	-	-
Leukotoxin E	*lukE*	-	-	-	-
Panton-Valentine leukocidin	*lukF-PV*	-	-	-	-
Panton-Valentine leukocidin	*lukS-PV*	-	-	-	-
Toxic shock syndrome toxin	*tsst*	-	-	-	-

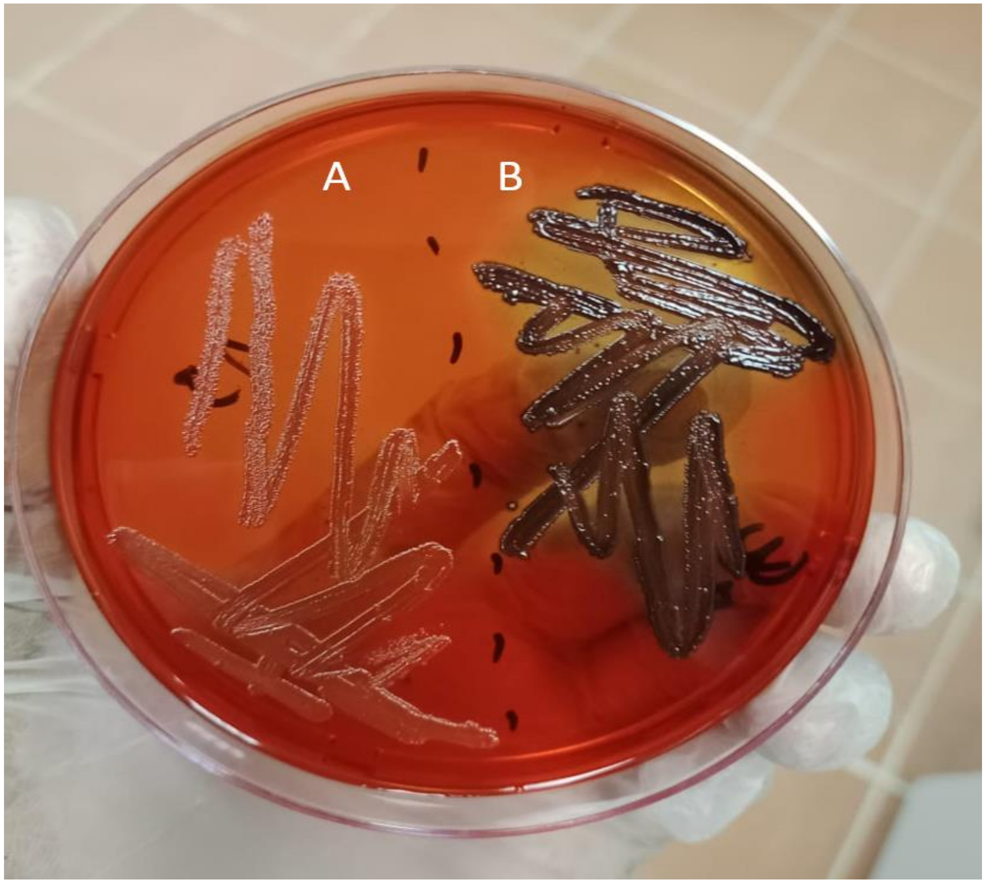

Figure S1. Congo red agar, showing biofim production, A. Biofilm negative isolate B. Biofilm positive isolate (9SE) showing the black metallic sheen colonies

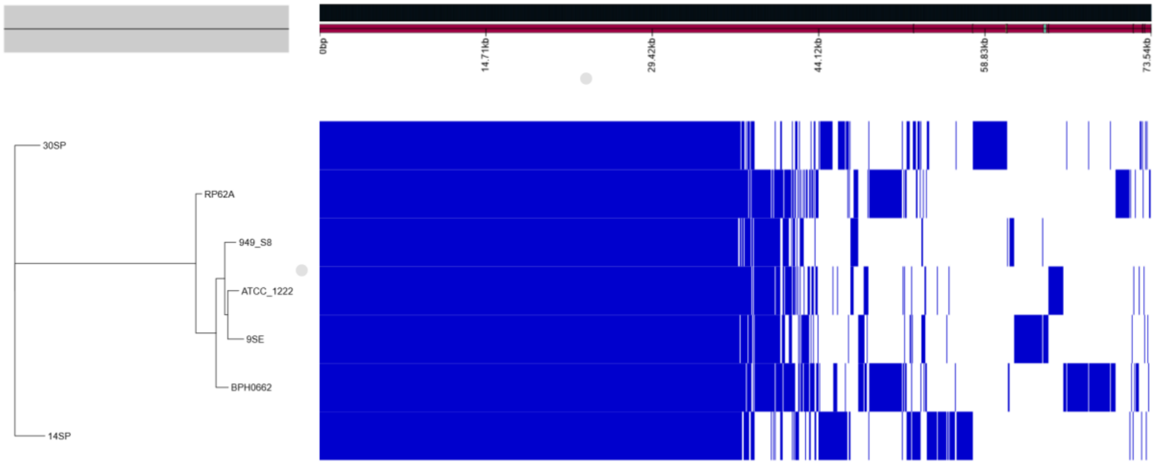

Figure S2: *S. epidermidis* variable genome. Only the variable genome is shown. Gene clusters present in all genomes or present in only a single genome are viewed. Gene presence is indicated by a blue bar.

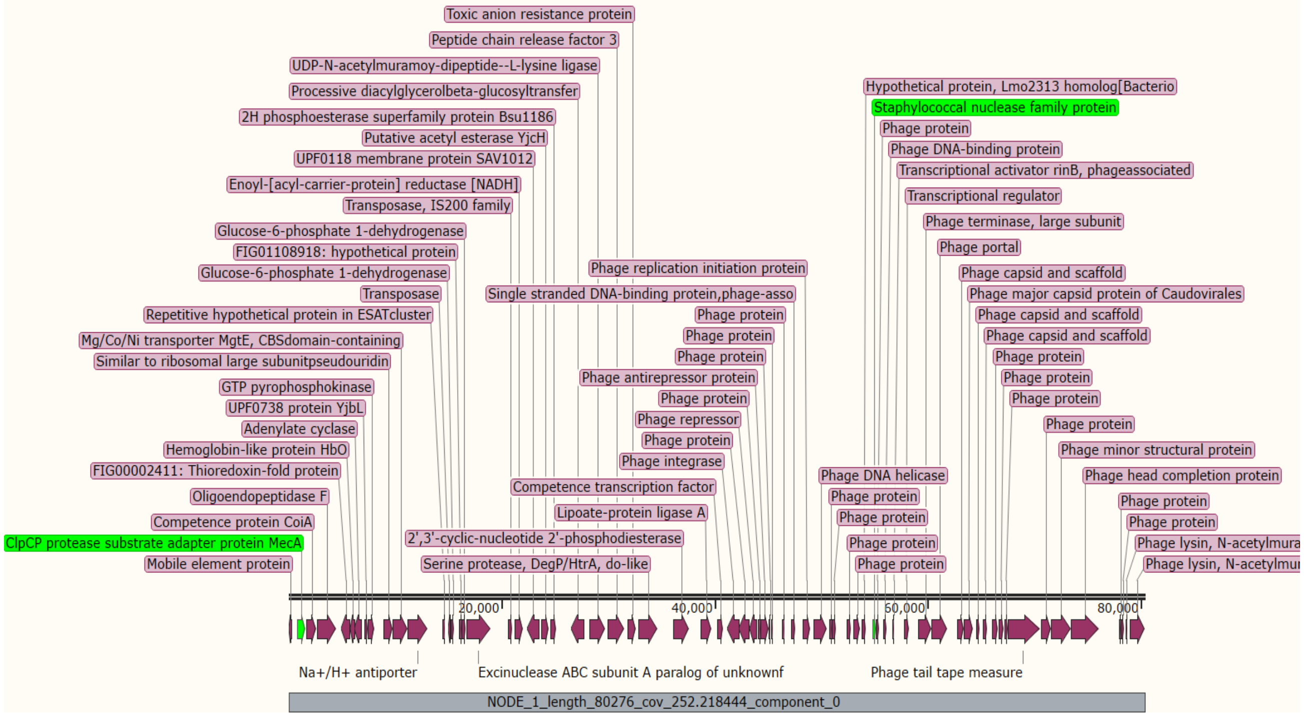

Figure S3. Linear representation of *S. epidermidis* (9SE) phage proteins, *Staphylococcal* nuclease family protein (green), mobile element proteins, transposases and *MecA* adaptor protein (green) identified clustered in contig one.

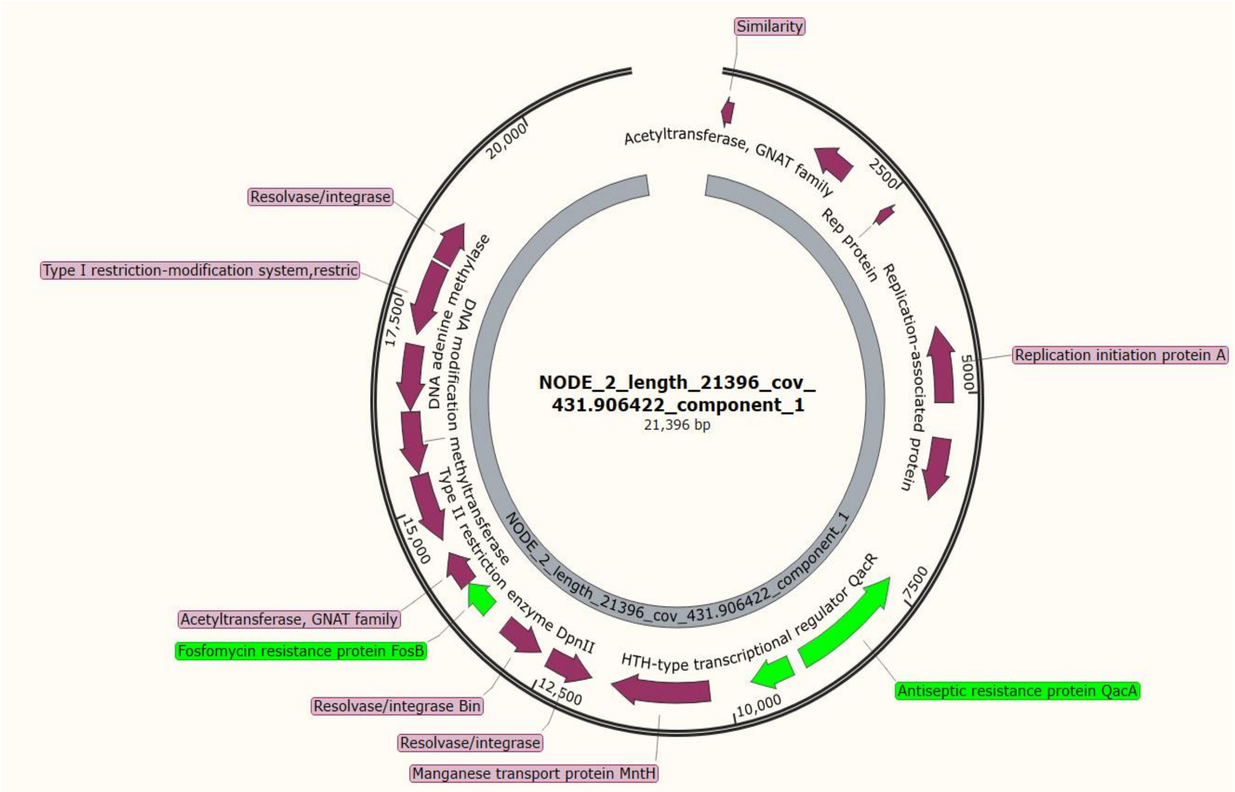

Figure S4. Circular representation of clustered ARGs (green) in isolate 9SE, the *repA* gene is shown in the end of the contig (5000bp).

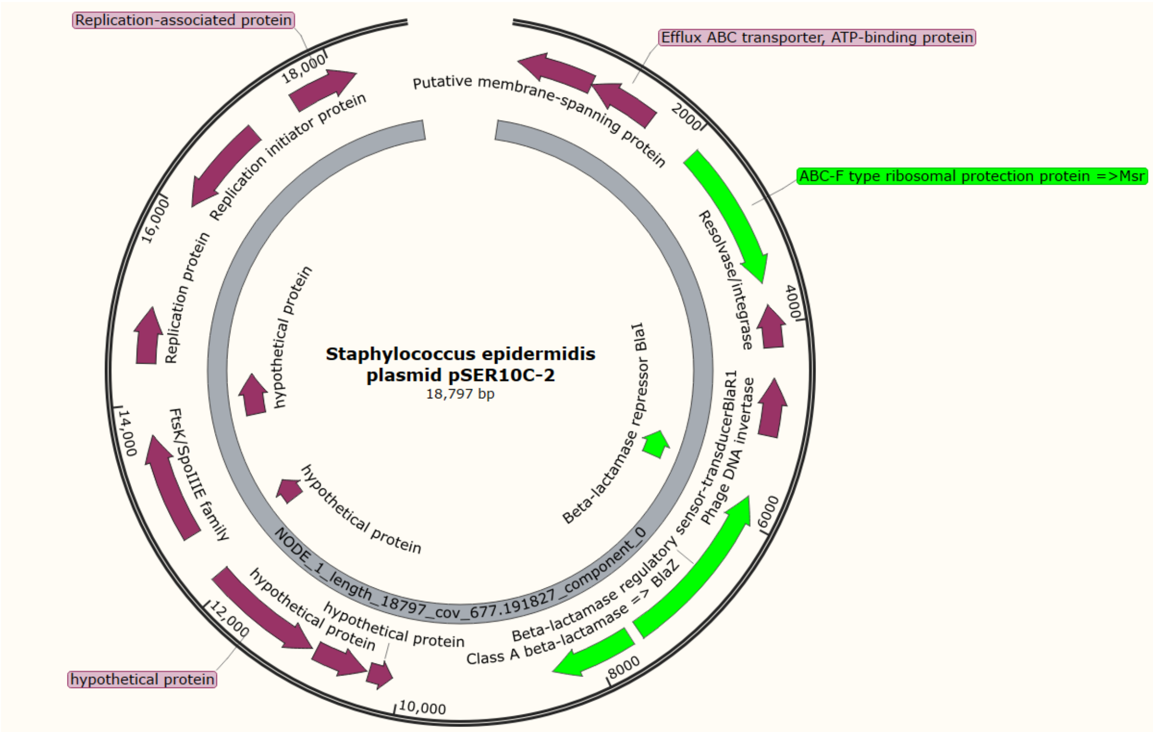

Figure S5. Circular representation of *S. epidermidis* (30SP) plasmid *p*SER10C-2, the identified ARGs are colored green
